# Simultaneous resection of coexisting pulmonary and mediastinal lesions by video-assisted thoracic surgery: a case-series study

**DOI:** 10.1186/s12893-022-01684-y

**Published:** 2022-06-20

**Authors:** Jiaheng Zhang, Yi Gao, Wenbing Zou, Wei Ping, Yunpeng Zhu, Xiangning Fu, Shengling Fu

**Affiliations:** 1 Department of Thoracic Surgery, Tongji Hospital, Tongji Medical College, Huazhong University of Science and Technology, Wuhan, 430030 Hubei People’s Republic of China; 2 The Second Clinical School, Tongji Medical College, Huazhong University of Science and Technology, Wuhan, 430030 Hubei People’s Republic of China

**Keywords:** Lung neoplasms, Mediastinal neoplasms, Simultaneous operation, Thoracic surgery, Video-assisted thoracoscopic surgery

## Abstract

**Background:**

With the growing number of patients with coexisting pulmonary and mediastinal lesions detected, reports about simultaneous video-assisted thoracic surgery (VATS) for these concurrent diseases are still rare. To further explore the safety and effectiveness of simultaneous resection of pulmonary and mediastinal lesions by uniportal or biportal VATS, we retrospectively analyzed the clinical data of the largest series of cases to date.

**Methods:**

From July 2018 to July 2021, all patients whose pulmonary lesions and mediastinal tumors were resected simultaneously in our institution were retrospectively reviewed. Their demographic and clinical data were collected and analyzed.

**Results:**

A total of 54 patients were enrolled, of whom 44 underwent unilateral uniportal VATS, 3 underwent bilateral uniportal VATS and 7 underwent unilateral biportal VATS. Seven cases were converted to thoracotomy during surgery. For the remaining 47 patients with various demographic and clinical characteristics, most of the operations were completed within 3 h (n = 33, 70.2%) with blood loss of no more than 100 mL (n = 43, 91.5%). The duration of chest tube drainage was 5.66 ± 3.34 days, and the average daily volume was 196.90 ± 122.31 mL. Four cases of postoperative complications occurred during hospitalization. The length of postoperative hospital stay was 8.60 ± 3.63 days. No severe complications or deaths were observed during follow-up.

**Conclusions:**

Uniportal and biportal VATS are safe and effective for simultaneous resection of selected coexisting pulmonary and mediastinal lesions, but the indications and operational details need more evaluation.

## Background

Lung cancer is a major health problem worldwide. Because of their anatomical proximity, the coexistence of pulmonary and mediastinal lesions has been detected more frequently with the development and popularization of medical imaging technology [1–7]. Correspondingly, more attention has been concentrated on the optimal measures to deal with these concurrent diseases and invasive lesions. Staged operations are available for some cases, but theoretically, combined resection could better reduce the financial burden on patients and avoid delays in treatment. For invasive lesions, open surgery is still preferred, but minimally invasive treatment should also be utilized as much as possible.

Video-assisted thoracic surgery (VATS) has been applied widely in the clinical diagnosis and treatment of thoracic diseases. Simultaneous thoracoscopic resection seems to be an ideal treatment option for coexisting pulmonary and mediastinal lesions, but convincing evidence is required and most of the studies thus far are small-sample and the types of patients are limited [4–6]. Our institution is one of the largest thoracic surgery centers in China with numerous operations performed every year, and has abundant surgical experience in uniportal VATS. Here, the aim of this study is to present the largest sample of uniportal VATS for simultaneous resection of pulmonary and mediastinal lesions and retrospectively analyze these data to help evaluate their feasibility and safety.

## Methods

### Study design and patient collection

Patients who underwent simultaneous operation for pulmonary and mediastinal lesions at the Department of Thoracic Surgery, Tongji Hospital, Tongji Medical College, Huazhong University of Science and Technology between July 2018 and July 2021 were reviewed. A total of 87 patients were identified, of whom 33 underwent thoracotomy and 54 underwent VATS. These 54 patients were analyzed in this study. (The other 33 cases were also reviewed for comparison and discussion, but their data are not listed directly.) Forty-four patients underwent unilateral uniportal VATS, 3 patients underwent bilateral uniportal VATS, and 7 patients underwent unilateral biportal VATS. Seven cases were converted to thoracotomy during surgery. Therefore, there were 47 cases of VATS performed successfully. Follow-up data were obtained through telephone interviews. All patients were followed up for 3–36 months (mean 12.5 months), with the exception of 2.

### Preoperative assessment

**Fig. 1:**
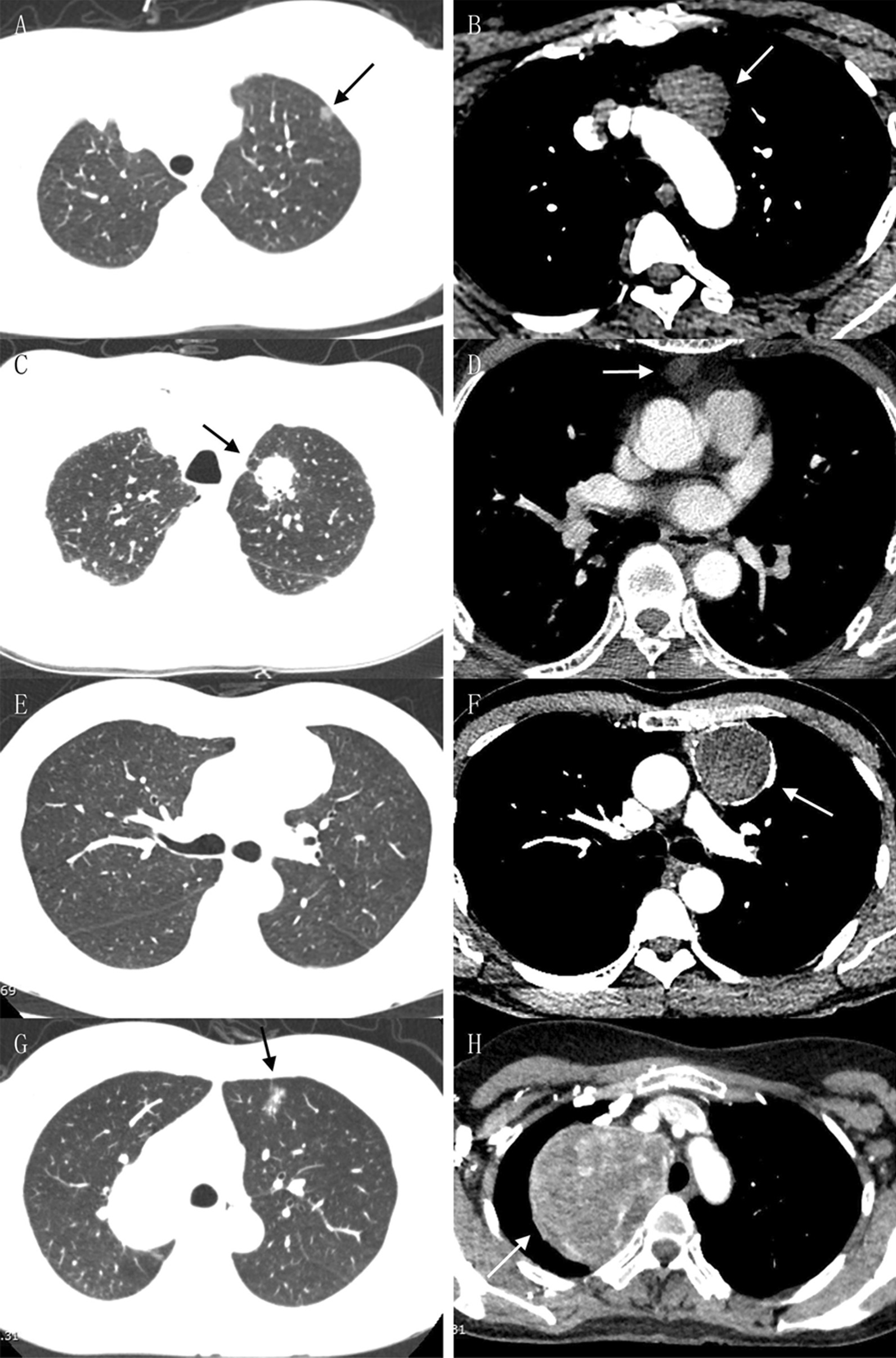
Chest CT images of representative patients. The arrows denote lesions. **A**, **B** A 10 mm left upper lobe GGO (microinvasive adenocarcinoma) associated with a 38 mm anterior mediastinal mass (hemangioma). **C**, **D** A 35 mm left upper lobe mass (invasive adenocarcinoma) associated with a 13 mm anterior mediastinal mass (bronchogenic cyst). **E**, **F** A 50 mm anterior mediastinal lesion (thymoma of type B2) invading surrounding lung tissues. **G**, **H** A 70 mm mediastinal mass (thyroid follicular neoplasm) and its coexisting 15 mm left upper lobe lesion (microinvasive adenocarcinoma), which were difficult for VATS and resected by thoracotomy

All patients underwent chest contrast-enhanced computed tomography (CT), from which the coexistence of pulmonary and mediastinal lesions was found and carefully evaluated (Fig. 1). Mediastinal lymph node enlargement was examined by enhanced chest CT as well, and some patients underwent mediastinal magnetic resonance imaging (MRI) to determine whether the mediastinal lesions were cystic or solid. Tumor markers of lung cancer and T-Spot test were used for differential diagnosis. To determine the presence of distant metastasis, head MRI, whole-body osteonuclide scans, and liver and adrenal gland ultrasonography were routinely performed, while positron emission tomography (PET)-CT was also used in a few cases. For patients suspected of myasthenia gravis (MG) with mediastinal tumors, electromyography (EMG) was performed routinely, and autoantibodies including Acetylcholine receptor (AChR) antibody, Muscle-specific kinase (MuSK) antibody and other antibodies were also detected for some patients. In addition to the typical clinical features of MG, patients with immunological, pharmacological, and/or neuroelectrophysiological manifestations can be clinically diagnosed as MG. Meanwhile, the patients received ultrasonography to check the conditions of their cardiovascular system and major organs, including the spleen, pancreas and kidney. Preoperative assessment also included an electrocardiogram, routine blood test, routine urine test, renal function test, blood gas analysis, and pulmonary function test to comprehensively evaluate the patients’ physiological status and tolerance for surgical treatment. Ground glass nodules (GGNs) were observed for at least three months and removed only when the nodules persisted or increased on subsequent imaging examination. Pulmonary nodules that may be inflammatory according to imaging were re-examined after anti-inflammatory treatment and removed when there was no reduction. Preoperative neoadjuvant therapy was performed for lung cancer that was clinically assessed as stage IIIA or above.

### Surgical technique

**Fig. 2:**
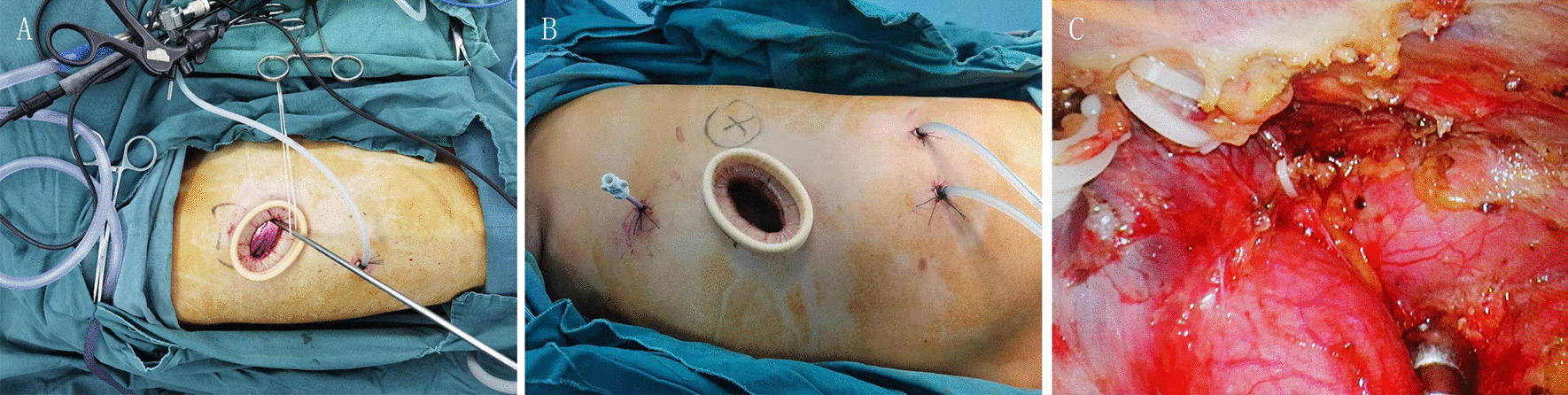
Intraoperative photos of uniportal VATS. **A** The surgical incision of uniportal VATS for the resection of the posterior segment of the right upper lobe and an anterior mediastinal mass. **B** The surgical incision of uniportal VATS for the resection of the posterior segment of the left upper lobe and an anterior mediastinal mass. **C** The ligation of the internal thoracic vein on the right-sided approach

General anesthesia was applied with double-lumen endotracheal intubation. For patients with coexisting lesions on the same side, unilateral uniportal VATS was performed while the patient was placed in the lateral decubitus position. After disinfection, a single incision of approximately 3 cm was made in the 5th intercostal space (ICS). The location of the incision was posterior to the midaxillary line (Fig. 2A, B). For biportal VATS, incisions were made in the 5th and 7th ICSs. Exploration was carried out for the locations, sizes and invasion of lesions, the quality of lungs, and the conditions of lung fissures. Meanwhile, pleural adhesions were separated carefully when found. If thoracoscopic separation of pleural adhesions was difficult, thoracoscopic surgery was converted to thoracotomy.

Resection of pulmonary lesions was usually performed first. For visible peripheral pulmonary nodules, wedge resection was performed for intraoperative frozen section examination. The surgical method including the type of pulmonary resection and whether lymph node dissection or sampling was necessary, was selected according to the results of intraoperative frozen section examination and preoperative examination. For patients requiring segmentectomy or lobectomy, anatomic resection was performed. After complete hemostasis, we examined air leakage of the lung. In some cases, the mediastinal lesions were excised first due to their large sizes or the surgeons’ personal preference. For the resection of mediastinal tumors, tissues in the area surrounded by the left innominate vein, the contralateral pleura, and the phrenic nerve on the surgical side were excised in one piece. For the right thoracic incision, the internal thoracic vein would be cut when it blocked the visual field and affected the exposure of the left innominate vein (Fig. 2C).

If two coexisting lesions were located on different sides, bilateral uniportal VATS was selected. The selection of surgical incision and surgical procedures were similar to uniportal VATS. For patients with MG, the mediastinal tumors were resected, and bilateral mediastinal fat clearance was performed by bilateral uniportal VATS.

For the lesions suspected to invade surrounding tissues (it was most common that mediastinal lesions invaded lung tissues), according to the tumor-free principle, the adjoined tissues invaded or attached tightly by the primary lesions were resected first. After all the surrounding tissues were separated, the primary lesion was finally removed.

### Data collection and analysis

Data including the demographic information, clinical manifestations, operative details, and perioperative outcomes were collected. The ASA category was established by the American Society of Anesthesiologists to classify patients undergoing anesthesia according to their physical conditions and the risk of surgery, which includes six levels from the best (1st) to the worst (6th). Data processing was performed using SPSS (ver. 20; SPSS, Inc., Chicago, IL, USA) for Windows. A descriptive analysis of the variables collected in this research was carried out.

## Results

The general characteristics of all 54 patients are summarized in Table 1. One patient had a history of coronavirus disease 2019 (COVID-19), and her operation and postoperative course were uneventful. The most common symptoms were cough (n = 7, 13.0%), chest tightness (n = 7, 13.0%) and chest pain (n = 6, 11.1%). Three patients had muscle weakness, two of whom were diagnosed with MG, while the remaining patient had ectopic adrenocorticotropic hormone (ACTH) syndrome. In sum, 31 patients had no associated symptoms and found thoracic lesions incidentally during routine examinations or diagnosis for diseases of other systems.

**Table 1:** General information of the patients

Variable	Mean ± SD (range) or no.
Gender (n)	
Men	26
Women	28
Age at diagnosis (year)	53.87 ± 11.58
Medical history (n)	
Hypertension	17
Diabetes	6
Pulmonary emphysema	3
Chronic bronchitis	3
COPD	1
Asthma	2
Coronary heart disease	5
Arrhythmia	4
Cerebral infarction	3
Thyroid cancer	2
Chest wall tuberculosis	1
COVID-19	1
History of surgery	25
Thoracic surgery	0
Smoking history (n)	16
ASA category (n)	
1th	5
2th	31
3th	18
Symptoms (n)	
Cough	7
Chest tightness	7
Chest pain	6
Expectoration	6
Dyspnea	4
Muscle weakness	3
Myasthenia gravis	2
Limb pain	2
Hemoptysis	1
Fever	1
Edema of lower limbs	1
Asymptomatic or no related symptoms	31

*ASA* American Society of Anesthesiologists, *COPD* chronic obstructive pulmonary disease, *COVID-19* Coronavirus Disease 2019

For the 35 patients whose pulmonary and mediastinal lesions were anatomically isolated and pathologically irrelevant to each other, Table 2 showed the pathological features of their pulmonary lesions, which included ground glass opacity (GGO), solid nodules, and masses associated with mediastinal lesions of varied sizes on imaging (Fig. 1A–D). Their anatomical locations included all five lung lobes, and the TNM stage of lung cancer ranged from 0 to IIIA. The only case of stage IIIA was a squamous cell carcinoma treated with radiotherapy and chemotherapy before surgery. Various pathological types were found in pulmonary benign lesions, with 3 cases of benign nodules unclassified pathologically. Table 3 shows the pathological features of the isolated mediastinal lesions. Most of them were located in the anterior mediastinum (n = 27, 77.1%). Benign cysts (n = 15, 42.9%) were the most common types among them, which included bronchogenic cysts, thymic cysts, and pericardial cysts. Almost half of these lesions (n = 16, 45.7%) were related to the thymus.

**Table 2:** Locations and pathological diagnosis of isolated pulmonary lesions

Variable	Mean ± SD (range) or no.
Lung cancer	21
Size (mm)	19.52 ± 12.32
Anatomical site (n)	
LUL	6
LLL	4
RUL	7
RLL	2
RML + RLL	2
Pathological type (n)	
Adenocarcinoma	18
AIS	3
MIA	4
ICA	11
Squamous cell carcinoma	1
Adenosquamous carcinoma	1
Lymphoepithelioma-like carcinoma	1
Stage (n)	
0	3
IA1	5
IA2	4
IA3	3
IB	4
IIB	1
IIIA	1
Pulmonary benign diseases	14
Anatomical site (n)	
LUL	2
LLL	2
RUL	3
RML	2
RLL	4
LUL + LLL	1
Pathological type (n)	
Chronic inflammation	4
Reactive lymph node hyperplasia	2
Tuberculosis	2
Sclerosing lung cell tumor	1
Lung cyst	1
Infectious pneumonia	1
Benign nodules	3

*AIS* adenocarcinoma in situ, *ICA* invasive adenocarcinoma, *LUL* left upper lobe, *LLL* left lower lobe, *MIA* minimally invasive adenocarcinoma, *RUL* right upper lobe, *RML* right middle lobe, *RLL* right lower lobe

**Table 3:** Locations and pathological diagnosis of isolated mediastinal lesions

Variable	Mean ± SD (range) or no.
Size (mm)	27.71 ± 16.39
Anatomical site (n)	
Upper	1
Anterior	27
Middle	1
Posterior	2
Upper anterior	4
Pathological type and stage (n)	
Thymoma	7
AB	3
B1 + B2	1
B2	3
Bronchogenic cyst	7
Thymic cyst	6
Pericardial cyst	2
Thymic carcinoma	2
Thymic hyperplasia	1
Lipoma	1
Hemangioma	1
Neuroendocrine tumor (ACTH)	1
Unclassified benign lesions	7

*ACTH* adrenocorticotropic hormone

Nineteen patients underwent simultaneous resection because the mediastinal tumors were suspected to invade the lung (n = 17, 89.5%), or vice versa (n = 2, 10.5%) (Fig. 1E, F). As shown in Table 4, the invasive lesions were much larger than the isolated ones mentioned above (51.29 ± 12.06 mm vs. 27.71 ± 16.39 mm for mediastinal lesions as example). It was no surprise that thymoma and thymic carcinoma constituted the majority of cases (n = 11, 57.9%), but there were also some lesions, such as bronchogenic cysts and pulmonary abscesses, found to be noninvasive after surgery, for which the surrounding tissues were resected due to the tight adhesion between them. Two cases of invasive lesions were located at both the anterior and posterior mediastinum. One of them was an anterior mediastinal thymic cyst and posterior mediastinal ganglioneuroma, and the other one was thymoma (type B2) shown as two lesions. Finally, 11 cases of invasion were confirmed by postoperative pathological examination.

**Table 4:** Pathological diagnosis of lesions suspected to invade surrounding tissues

Variable	Mean ± SD (range) or no.
Sizes of primary lesions (mm)	50.95 ± 12.75
Anatomical sites of primary lesions (n)	
Mediastinum	17
Anterior	12
Posterior	1
Upper anterior	2
Anterior + Posterior	2
Lung	2
LUL	1
LLL	1
Pathological type (n)	
Thymoma	8
B1	2
B1 + B2	1
B2	4
B3	1
Thymic carcinoma	3
Hodgkin lymphoma	2
Thymic Cyst + Ganglioneuroma	1
Tuberculosis	1
Bronchogenic cyst	1
Sclerosing lung cell tumor	1
Pulmonary abscess	1
Mature teratoma	1
Invaded site (n)	
Mediastinum	2
Anterior	1
Posterior	1
Lung	17
LUL	6
LLL	1
RUL	6
RLL	3
LLL + LUL	1
Invasion confirmed by biopsy (n)	11

*LUL* left upper lobe, *LLL* left lower lobe, *RUL* right upper lobe, *RML* right middle lobe, *RLL* right lower lobe

Surgical details and intraoperative characteristics are stated in Table 5. Seven cases of VATS were converted to thoracotomy. Two cases of conversion were because of the severe pleural adhesions, and 2 cases were respectively due to the massive invasion on lung or pericardium. The other 3 operations were converted on account of the large sizes and severe local invasion of mediastinal lesions. These cases were converted to thoracotomy just after exploration by VATS. For the remaining 47 non-converted VATS, 38 patients had pulmonary lesions resected first. Coincidentally, all 3 cases of bilateral uniportal VATS (including the one converted to thoracotomy) had resection of the mediastinal lesions first. The only frequent intraoperative unplanned events were pleural adhesions (n = 18, 38.3%), which could usually be resolved without much difficulty. Most of the operations were completed within 3 h (n = 33, 70.2%). No heavy bleeding occurred, and most cases (n = 43, 91.5%) had blood loss of no more than 100 mL during surgery. A few cases of large mediastinal tumors (50 mm, 70 and 78 mm) resulted in more bleeding (300 mL, 300 mL, and 800 mL, respectively) due to their abundant blood supply.

**Table 5:** Operative details and characteristics

Variable	Mean ± SD (range) or no.
Surgical approach (n)	
Unilateral uniportal VATS	44
Converted to thoracotomy	5
Left side	16
Right side	23
Bilateral uniportal VATS	3
Converted to thoracotomy	1
Unilateral biportal VATS	7
Converted to thoracotomy	1
Left side	3
Right side	3
For the 47 cases of non-converted VATS	
Operational sequence (n)	
Lung first	38
Mediastinum first	9
Type of pulmonary surgery (n)	
Lobectomy	12
Double lobectomy	2
Segmentectomy	8
Wedge resection	27
Lymph node dissection (n)	11
Number of lymph nodes excised	20.00 ± 5.74
Groups of mediastinal lymph nodes removed (n)	
Left side	
4L	1
5	3
6	3
7	4
8	1
9	4
Right side	
2R	7
4R	7
7	7
9	5
Operation time (min)	167.83 ± 69.22
Intraoperative blood loss (mL)	57.55 ± 128.33
Range (n)	
≤ 100	43
> 100	4
Intraoperative unplanned events (n)	
Pleural adhesions	18

After thoracoscopic surgery (Table 6), patients with MG or major surgery were taken back to the intensive care unit (ICU) with tracheal intubation for ventilator-assisted breathing. One patient was repeatedly put on ventilators, with the overall duration of 285 h, which was caused by a myasthenic crisis. There were 3 other cases of postoperative complications. One of them presented acutely with convulsions, ventricular fibrillation, hypertension, and hypoxemia, which was related to the patient’s primary disease-induced ectopic ACTH syndrome and was eventually controlled. The other two patients had massive pleural effusion cured by thoracentesis. The 47 patients left the hospital 8.60 ± 3.63 days (range 5–23 days) after surgery with no perioperative mortality. One patient reported bronchospasm after discharge, which is one of the common postoperative pulmonary complications (PPCs) [8–10]. No recurrence or death occurred during follow-up.

**Table 6:** General postoperative conditions of the 47 cases of non-converted VATS

Variable	Mean ± SD (range) or no.
Postoperative mechanical ventilation (n)	7
≤ 24 h	5
> 24 h	2
Duration of chest tube drainage (d)	5.66 ± 3.34
Average daily volume of drainage (mL/d)	196.90 ± 122.31
Postoperative hospital stay (d)	8.60 ± 3.63
Postoperative complications (n)	4
Cardiovascular dysfunction + convulsions	1
Myasthenic crisis	1
Massive pleural effusion	2
Perioperative mortality (n)	0
Postoperative adjuvant therapy (n)	
Chemotherapy	7
Radiotherapy	3
Chemotherapy + Radiotherapy	2
Targeted therapy	1
Immunotherapy	1
Complications reported during follow-up (n)	
Bronchospasm	1

## Discussion

Yoon et al. indicated that the expected prevalence of anterior mediastinal nodular lesions is approximately 1% in populations aged 55–74 at high risk for lung cancer during low-dose chest CT screening [11]. Similarly, combined surgery for coexisting pulmonary and mediastinal lesions accounted for approximately 0.93% of the pulmonary operations between July 2018 and July 2021 in our hospital and 6.64% of the mediastinal operations. Although the mechanism has not been clarified, thymoma is associated with an increased risk of a secondary neoplasm, such as primary lung cancer [12, 13]. With the help of rapidly developed medical imaging technology and regular examinations before thoracic surgery, coexisting pulmonary and mediastinal lesions are being increasingly detected clinically, but their management remains a challenge due to the lack of surgical guidelines or reports of mass cases.

The adjacent anatomical locations of the lung and mediastinum allow the possibility of combined resection, and some surgeons have already tried this approach and shared their valuable experience [1–7]. As a minimally invasive surgery, VATS has been applied widely in the diagnosis and treatment of thoracic diseases. Since first introduced by Migliore in 2001, uniportal VATS has been increasingly adopted by virtue of its advantages, such as a smaller incision, less intraoperative bleeding, rapider recovery, and a shorter duration of surgery, drainage, and hospitalization than multiportal VATS [14–17]. Taken together, VATS, especially uniportal VATS, seems to be an optimal choice for coexisting pulmonary and mediastinal lesions, but the potential risks from complex techniques and prolonged surgery make it necessary for more assessment of its feasibility and safety.

For the 54 cases enrolled in this study, simultaneous VATS was successfully performed in 47. Most of the non-converted VATS were completed within 3 h (n = 33, 70.2%) and with bleeding no more than 100 mL (n = 43, 91.5%), which is comparable to the data shown in other studies [4–6]. Specifically, 12 cases (25.5%) of combined surgery were completed within 2 h, 29 cases (61.7%) were between 2 and 4 h, and 6 cases (12.8%) took more than 4 h. The pleural adhesions, large sizes of lesions, and dissection of lymph nodes and invaded tissues were the main causes of prolonged surgical duration. Besides severe pleural adhesions, another factor that converted VATS into thoracotomy was the large size (54.67 ± 14.99 mm vs. 32.87 ± 17.95 mm) or extensive invasion of the mediastinal lesions, especially when the number of local metastases was more than two. In addition to the surgeons’ habits, biportal VATS was usually chosen instead of uniportal VATS when both coexisting lesions were relatively large but had no severe local invasion.

**Fig. 3:**
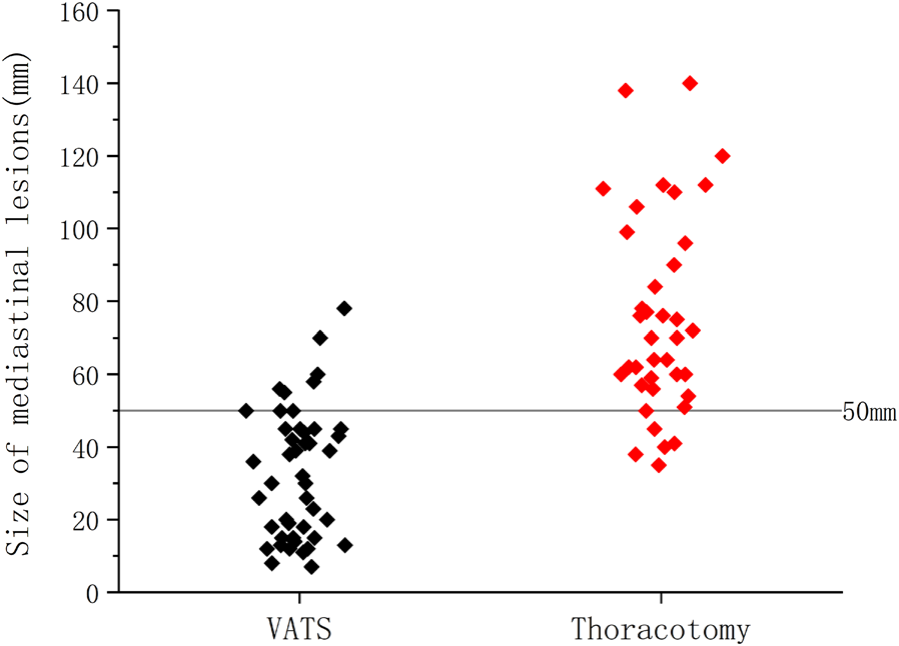
Sizes of mediastinal lesions between VATS and thoracotomy

To ensure that all lesions are resected, median sternotomy is generally chosen for invasive thymoma [18]. We gathered the data of combined thoracotomy as well, from which it could be found that the average size of the mediastinal lesions was 79.91 ± 26.99 mm, and almost all of them (84.8%) had invaded the surrounding lung tissues (Fig. 1G, H). As shown in Fig. 3, a diameter of 50 mm for mediastinal lesions can be chosen as a turning point by comparing the data between successful VATS and open surgery (including the operations converted to thoracotomy). Therefore, for mediastinal lesions no larger than 50 mm coexisting with pulmonary lesions, if there is no severe local invasion on imaging, it will be feasible to perform uniportal VATS exploration first and decide whether to continue thoracoscopic resection or convert to thoracotomy.

For the resection of pulmonary lesions, the incision should be located in the midaxillary line, slightly anterior for segmental resection near the anterior hilum and slightly posterior for segmental resection near the posterior hilum. For combined VATS, because the location of the single port needed to take both the pulmonary and mediastinal lesions into account, the operation was more difficult. It is necessary to choose the location of the surgical incision with flexibly, and typically a single incision is made in the midaxillary line of the 5th ICS near the posterior axillary line. For the right thoracic incision, if the incision is slightly anterior, the internal thoracic vein will block the visual field and affect the exposure of the left innominate vein. In this case, the internal thoracic vein can be cut (Fig. 2C). For left thoracic incision, surgical instruments are susceptible to interference from the heart if the incision is too far posterior, and resection of segments or lobes at certain sites may not be easy. None of our patients who underwent uniportal VATS had a thoracotomy or auxiliary port added because of poor placement of the incision.

It seems that our drainage duration (5.66 ± 3.34 days) was longer than that in other studies, but the difference in standards for the removal of chest tubes should not be ignored. In our department, the last 24-h drainage volume should be less than 200 mL. Most cases of massive drainage occurred after systematic lymph node dissection, and the other case of pleural effusion was mostly due to the large inner wound surface left by a 78 mm mediastinal lesion. All of them were resolved by conservative treatment. Besides, the standards for discharge also delayed some patients’ leaving. The chest imaging (X-ray photograph or CT) and routine blood test (especially the WBC count and neutrophil proportion) were required to become normal before discharge, otherwise the patients should stay to receive further observation and treatment.

Two cases of severe postoperative complications occurred during hospitalization. One case of ectopic ACTH syndrome broke out acutely 3 days after uniportal VATS. Fortunately, the condition gradually became controlled, and the patient left the hospital 15 days after surgery. The other case showed difficultly weaning from mechanical ventilation. Although MG had not been diagnosed before surgery, the cause was discovered to be postoperative myasthenic crisis (PMC). The patient recovered and left the hospital 23 days after surgery. In sum, these severe complications were mainly caused by the primary diseases of the patients rather than the operations.

Telephone follow-up was performed for all 47 non-converted VATS cases, and 2 were lost to follow-up. One case of new-onset bronchospasm and associated paroxysmal hypoxemia was reported. According to the Assess Respiratory Risk in Surgical Patients in Catalonia (ARISCAT) score, this case of long-duration (290 min) intrathoracic surgery can be leveled as high risk for PPCs [8, 9]. For combined surgery, relative extension of operation time and trauma is inevitable, but the risk remains controllable. Other complaints reported during follow-up included pain, cough, and small amounts of pleural effusion, which were gradually improved over time.

Consistent with normal thoracoscopic operations, combined uniportal or biportal VATS does not have many absolute contraindications. Besides enough physical toleration and relatively early clinical stages required by most thoracic tumor operations, coexisting lesions that are difficult to be resected through the same surgical incision might be a relative contraindication, although the clinical judgment lacks quantitative criteria and needs abundant operational experience. The history of thoracic surgery should also be taken into account in view of the fact that severe pleural adhesion is one of the main factors that turned VATS into thoracotomy.

It is worth discussing whether pulmonary lesions or mediastinal tumors should be removed first. Normally, surgeons tend to first resect the pulmonary lesions [4–6], which is the same in our study (38:9). Technically, the resection of pulmonary lesions can provide more space for the exposure of mediastinal lesions. When the mediastinal lesions are large but have not invaded surrounding tissues, mediastinum-first resection is preferred. However, if mediastinal tumor invades or adheres closely to the surrounding tissues, the invaded tissues should be removed first. In general, lung-first VATS helps to maintain a clear field of vision for both pulmonary and mediastinal surgery. If mediastinal lesions are treated first before pulmonary lesions, the exudation of mediastinal surgical wounds will flow to the lung, thus affecting the clear field of pulmonary surgery.

Although the patients enrolled in this study were various, its reliability is limited by the retrospective design and relatively small sample size. Items such as the incision length were not analyzed due to the missing of records, and it failed to establish a case-control study since there lacked enough comparable cases of multiportal VATS or staged operations for coexisting pulmonary and mediastinal lesions in our hospital. We tried to assess the indications by comparing the characteristics of cases between VATS and thoracotomy, but this was inevitably influenced by the subjective bias and habits of surgeons.

In brief, this is thus far the largest case series analysis about combined resection of pulmonary and mediastinal lesions through uniportal VATS. We analyzed the clinical pathological characteristics of these patients and the efficacy and safety of the operations. The surgical indications and selection of the appropriate incision site were also explored. The reliability of this study is limited due to the small number of cases and non-comparative design. Further researches with larger sample sizes are needed.

## Conclusions

We found that simultaneous resection of pulmonary and mediastinal lesions by an experienced thoracic surgeon using uniportal or biportal VATS is safe and feasible. This surgical method has certain indications: in general, mediastinal lesions that are less than 50 mm without serious local invasion, and pulmonary lesions that are in the early or middle stages. In addition, the importance of appropriate surgical procedures is noteworthy.

## Data Availability

The datasets used and/or analyzed during the current study are available from the corresponding author on reasonable request.

## Abbreviations

AChR: Acetylcholine receptor
ACTH: Adrenocorticotropic hormone
ARISCAT: Assess Respiratory Risk in Surgical Patients in Catalonia
ASA: American Society of Anesthesiologists
COVID-19: Coronavirus disease 2019
CT: Computed tomography
EMG: Electromyography
GGN: Ground glass nodule
GGO: Ground glass opacity
ICS: Intercostal space
ICU: Intensive care unit
MG: Myasthenia gravis
MRI: Magnetic resonance imaging
MuSK: Muscle-specific kinase
PET: Positron emission tomography
PMC: Postoperative myasthenic crisis
PPC: Postoperative pulmonary complication
VATS: Video-assisted thoracic surgery
